# Supplementary material for: “My Friends Would Believe My Word”: Appropriateness and Acceptability of Respondent-Driven Sampling in Recruiting Young Tertiary Student Men Who Have Sex with Men for HIV/STI Research in Nairobi, Kenya
Source: Int J Environ Res Public Health. 2022 Jun 15;19(12):7331. doi: 10.3390/ijerph19127331 (PMC9223518; doi:10.3390/ijerph19127331)
Supplement: Supplementary file 1 [file ijerph-19-07331-s001.zip › ijerph-1621663-supplementary.pdf]

File S1: COREQ Checklist

Number	Where reported in the manuscript
**Domain 1: Research team and reflexivity**	
Personal characteristics	
1. Interviewer	Data collection
2. Credentials	Title page (link to ORCiD)
3. Occupation	Title page (link to ORCiD)
4. Gender	Title page (link to ORCiD)
5. Experience and training	Title page (link to ORCiD)
Relationship with participants	
6. Relationship established	Data collection
7. Participant knowledge of the interviewer	Data collection
8. Interviewer characteristics	Data collection
**Domain 2: Study design**	
Theoretical framework	
9. Methodological orientation and theory	Theoretical framework
Participant selection	
10. Sampling	Participants
11. Method of approach	Participants
12. Sample size	Participants
13. Non-participation	Participants
Setting	
14. Setting of data collection	Data collection
15. Presence of non-participants	Data collection
16. Description of sample	Participants and results
Data collection	
17. Interview guide	Data collection
18. Repeat interviews	N/A
19. Audio/visual recording	Data collection
20. Field notes	Data collection
21. Duration	Data collection
22. Data saturation	Data collection
23. Transcripts returned	N/A
**Domain 3: Analysis and findings**	
Data analysis	
24. Number of data coders	Data analysis
25. Description of the coding tree	Results
26. Derivation of themes	Data analysis
27. Software	Data analysis
28. Participant checking	Trustworthiness of the data
Reporting	
29. Quotations presented	Results
30. Data and findings consistent	Results
31. Clarity of major themes	Results
32. Clarity of minor themes	N/A

File S2: Guide for interviews with service providers

Exploring the appropriateness, acceptability and challenges of RDS in recruiting TSMSM into a prospective HIV/STI bio-behavioral survey

Introduction: I would like to thank you for agreeing to participate in this interview. We are going to talk about a study we are planning to carry out on HIV and STI among TSMSM. We would like to hear your views about the methods we are thinking of using in this study so that you can let us know if the methods are suitable or not, and what we can do to improve them. Please feel free to engage in this conversation.

**Themes**	**Questions**	**Probes/prompts**	**Notes**
I would like to start by asking you about what you do in this organization:			
Role in the organization	Tell me about your role in this organization:	What do you do on a day-to-day basis?<br><br>What else do you do apart from what you have told me?	
	What type of contact do you have with MSM clients?	How often do you work with MSM? Every day? Occasionally? Rarely?<br><br>Are some of the MSM you see tertiary/college/university students?<br><br>If so, how often do you work with these TSMSM?	
Now, I would like to ask you about the type of services offered by your organization:			
Services offered by the organization	What type of services does your organization provide?	HIV/STI awareness? Prevention? Treatment?<br><br>Psychosocial support?	
	Apart from what your organization offers, what other services exist for MSM in and around Nairobi?	HIV/STI awareness? Prevention? Treatment?<br><br>Psychosocial support?<br><br>Which organizations offer these services?	
	Who are the key population for your services?	MSM? Sex workers? Injecting drug users? Adolescents and young women?	
	Approximately how many MSM did your organization serve this	Of these, approximately how many/ what proportion were TSMSM?	

	past year (last 12 months)?	In your day-to-day record keeping, do you have a way of documenting an MSM client's level of education?	
Now I would like you to help me understand the MSM community/networks in and around Nairobi:			
MSM community and networks	Think about MSM in and around Nairobi. How can you describe them?	Could you describe for me the different subgroups of MSM?<br><br>Do they differ by: Age; Education; Income; Ethnicity; Gender identity; Sexual preferences; Other?<br><br>How much interaction or contact is there between the sub-groups?<br><br>How do TSMSM interact with the different sub-groups that you just described?	
Now, I would like to describe to you how we plan to recruit TSMSM into the HIV/STI study. I will then ask you some questions that will help us plan the study better.<br><br>We are thinking of choosing a few TSMSM who are well connected and regarded in the TSMSM community. We will give them some invitation coupons that they could give to their peers to invite them to participate in the study. For every friend/peer they recruit into the survey, they will receive a cash incentive on top of the cash incentive they receive for participating themselves. The participants that come in will then be given coupons that they can use to recruit their friends/peers. The process will continue until we reach our sample size of 250 TSMSM. *[SHOW participant a sample of the invitation coupon and explain how it is used*].			
Appropriateness and acceptability of RDS	How well do you think this method would work in recruiting TSMSM into the study?	Is it a good method? Why?<br><br>Is it a bad method? Why?	
	Do you think TSMSM would be willing to invite other TSMSM using the invitation coupons?	What would make them to be willing?<br><br>What would make them to be unwilling?	
	After receiving an invitation coupon, how long do you think TSMSM would take to	1,2,3 days? A week? 2 weeks? 1 month?<br><br>What factors would influence how fast TSMSM come to	

	come in and participate in the study?	participate after receiving the coupon? COVID-19 restrictions? Schedule of classes? Examinations? Transport to the study site?	
	Approximately how long do you think it would take us to recruit 250 TSMSM into the study?	1,2 or 3 months? 6 months? 1 year?<br>What factors would influence how long it would take us to achieve our sample size?<br>COVID-19? Incentives? Schedule of classes? Examinations?	
RDS challenges and proposed solutions	What challenges are we likely to encounter when using this method of recruitment?	Failure to get participants?<br>Participants who are not MSM?<br>MSM who are not tertiary students?	
	How do you suggest we could address these challenges?	Extra incentives?<br>Screening to ensure participants are MSM?<br>Use of student IDS?	

Conclusion: Do you have any questions about the study before we end? Is there anything you would like to add to our discussion? [*TAKE time to address all questions and concerns*].

Thank you very much for taking time to talk to us. The information you have provided us with is important and will help us in planning for the study.

File S3: Guide for interviews with TSMSM
Exploring the appropriateness, acceptability and challenges of RDS in recruiting TSMSM into a prospective HIV/STI bio-behavioral survey

Introduction: I would like to thank you for agreeing to participate in this interview. We are going to talk about a study we are planning to carry out on HIV and STI among TSMSM. We would like to hear your views about the methods we are thinking of using in this study so that you can let us know if the methods are suitable or not, and what we can do to improve them. Please feel free to engage in this conversation.

Themes	Questions	Probes/prompts	Notes
I would like to start by asking you about your friends.			
Social networks	Tell me about your closest friends:	Are your friends also TSMSM?<br><br>Where do they study? Same institution as you or different one(s)?<br><br>Do you have friends who are not TSMSM? How would you describe them?	
	How many TSMSM do you know by name, and they know you by name, and they live and study in and around the city of Nairobi?	How many of these have you seen in the past one month?<br><br>How about in the past one week?<br><br>Have the COVID-19 restrictions affected how often you see your friends who are TSMSM? Do you see them more or less often?	
	How easy is it for you to contact your TSMSM friends?	Easy? Difficult? Not easy not difficult?	
	How do you contact your TSMSM friends?	Phone calls? Text messages? Social media (WhatsApp, Telegram, Instagram, Twitter)?	
Now, I would like to describe to you how we plan to recruit TSMSM into the HIV/STI study. I will then ask you some questions that will help us plan the study better.<br><br>We are thinking of choosing a few TSMSM who are well connected and regarded in the TSMSM community. We will give them some invitation coupons that they could give to their peers to invite them to participate in the study. For every friend/peer they recruit into the survey, they will receive a cash incentive on top of the cash incentive they receive for participating			

themselves. The participants that come in will then be given coupons that they can use to recruit their friends/peers. The process will continue until we reach our sample size of 250 TSMSM. *[SHOW participant a sample of the invitation coupon and explain how it is used*].			
Appropriateness and acceptability of RDS	How well do you think this method would work in recruiting TSMSM into the study?	Is it a good method? Why?<br><br>Is it a bad method? Why?	
	If you are selected and given say 3 coupons, how long would it take you to get 3 of your friends/peers to give them the coupons?	1,2,3 days? A week? 2 weeks? 1 month?	
	After giving a friend an invitation coupon, how long do you think they would take to come in and participate in the study?	1,2,3 days? A week? 2 weeks? 1 month?<br><br>What factors would influence how fast a friend comes to participate after receiving the coupon? COVID-19 restrictions? Schedule of classes? Examinations? Transport to the study site?	
	Do you think your friends would be willing to recruit their friends using such coupons?	What would make them to be willing?<br><br>What would make them to be unwilling?	
	Approximately how long do you think it would take us to recruit 250 TSMSM into the study?	1,2 or 3 months? 6 months? 1 year?<br><br>What factors would influence how long it would take us to achieve our sample size? COVID-19? Incentives? Schedule of classes? Examinations?	
RDS challenges and proposed solutions	What challenges are we likely to encounter when using this method of recruitment?	Failure to get participants?<br><br>Participants who are not MSM?<br><br>MSM who are not tertiary students?	

	How do you suggest we could address these challenges?	Extra incentives?<br><br>Screening to ensure participants are MSM?<br><br>Use of student IDS?	

Conclusion: Do you have any questions about the study before we end? Is there anything you would like to add to our discussion? [*TAKE time to address all questions and concerns*].

Thank you very much for taking time to talk to us. The information you have provided us with is important and will help us in planning for the study.
